# Venous thromboembolism prevention in intracerebral hemorrhage: A systematic review and network meta-analysis

**DOI:** 10.1371/journal.pone.0234957

**Published:** 2020-06-24

**Authors:** Vignan Yogendrakumar, Ronda Lun, Faizan Khan, Kristin Salottolo, Karine Lacut, Catriona Graham, Martin Dennis, Brian Hutton, Philip S. Wells, Dean Fergusson, Dar Dowlatshahi

**Affiliations:** 1 Division of Neurology, Department of Medicine, University of Ottawa, Ottawa, Canada; 2 Clinical Epidemiology Program, Ottawa Hospital Research Institute, Ottawa, Canada; 3 Trauma Research Department, St. Anthony Hospital, Lakewood, Colorado, United States of America; 4 EA3878, Université de Bretagne Occidentale, Brest, France; 5 Wellcome Trust Clinical Research Facility, Western General Hospital, Edinburgh, Scotland, United Kingdom; 6 Centre for Clinical Brain Sciences, University of Edinburgh, Edinburgh, Scotland, United Kingdom; 7 Division of Hematology, Department of Medicine, University of Ottawa, Ottawa, Canada; Maastricht University Medical Center, NETHERLANDS

## Abstract

**Introduction:**

To summarize and compare the effectiveness of pharmacological thromboprophylaxis to pneumatic compression devices (PCD) for the prevention of venous thromboembolism in patients with acute intracerebral hemorrhage.

**Methods:**

MEDLINE, PUBMED, EMBASE, and CENTRAL were systematically searched to identify randomized and non-randomized studies that compared each intervention directly to each other or against a common control (hydration, anti-platelet agents, stockings) in adults with acute spontaneous intracerebral hemorrhage. Two investigators independently screened the studies, extracted data, and appraised risk of bias. Studies with a high risk of bias were excluded from our final analysis. The primary outcome was the occurrence of venous thromboembolism (proximal deep vein thrombosis or pulmonary embolism) in the first 30 days.

**Results:**

8,739 articles were screened; four articles, all randomized control trials, met eligibility criteria. Bayesian network meta-analysis was performed to calculate risk estimates using both fixed and random effects analyses. 607 patients were included in the network analysis. PCD were associated with a significant decrease in venous thromboembolism compared to control (OR: 0.43, 95% Credible Limits [CrI]: 0.23–0.80). We did not find evidence of statistically significant differences between pharmacological thromboprophylaxis and control (OR: 0.93, 95% CrI: 0.19–4.37) or between PCD and pharmacological thromboprophylaxis (OR: 0.47, 95% CrI: 0.09–2.54).

**Conclusion:**

PCDs are superior to control interventions, but meaningful comparisons with pharmacotherapy are not possible due to a lack of data. This requires further exploration via large pragmatic clinical trials

**Trial registration:**

PROSPERO: CRD42018090960

## Introduction

Venous thromboembolism is a common complication for patients suffering from a spontaneous intracerebral hemorrhage (ICH) [1,2]. The two primary methods of VTE prevention in use today are mechanical compression and pharmacological thromboprophylaxis.

Mechanical prevention, in the form of compression stockings and hoses, graduated compression stockings, and pneumatic compression devices (PCD) have been used to varying degrees in clinical practice. Of the three, PCD have emerged as the most effective in VTE prevention [3], with the two former devices no longer recommended as first line therapy by international guidelines [4–7]. The use of PCD in the context of ICH is increasing in clinical practice [8]. However, trials displaying its effectiveness are limited and throughout the literature are concerns regarding compliance and cost [9,10].

While commonly used to prevent VTE in a variety of medical scenarios [11], the use of pharmacological thromboprophylaxis in ICH is an area of ongoing debate. The primary concern is that these medications may exacerbate intracranial bleeding but this has not been demonstrated in observational studies [1,12].

In this study, we sought to compare the effectiveness of each intervention indirectly through the use of network meta-analysis, which can inform comparisons between interventions based upon all available direct and indirect evidence [13].

## Methods

### Protocol and registration

The protocol for this study was registered with PROSPERO (CRD42018090960) and published [14]. This study was conducted using recommendations from the Cochrane Handbook for Systematic Reviews, and findings were prepared with guidance from the PRISMA extension statement for network meta-analysis [15]. The authors declare that all supporting data are available within the article and online-only supplement. As new patient data was not collected for this study, local research ethics board approval was not required.

### Eligibility criteria

Included studies were randomized controlled trials or non-randomized studies with a control group that enrolled adults (≥ 18 years) with acute spontaneous ICH who were treated with PCD or pharmacological therapy. Studies exclusively focused on subarachnoid or subdural hemorrhage, or traumatic etiologies were excluded. Studies that involved a mixed population (e.g. intensive care unit population) of patients were included if ICH patients were assessed separately within the study publication.

All studies investigating short-term pharmacotherapy were considered. No restrictions on dose, frequency, duration, or route of administration were used. We limited our inclusion to devices that provided PCDs only. We had no restrictions on whole leg vs. calf-only compression. Control populations were defined as those who did not receive PCD of pharmacological prophylaxis. These populations may receive intravenous hydration, antiplatelets, compression/graduated stockings, or any combination of the aforementioned.

### Information sources and trial search

We conducted a comprehensive search of MEDLINE, EMBASE, PubMed, and CENTRAL from inception until March 1^st^, 2018, as per our protocol, and performed an updated search from March 1^st^, 2018 to May 6^th^, 2020 via MEDLINE and EMBASE. A sample search strategy is available in S1 Table. Electronic searches were supplemented by hand searching of reference lists of relevant articles and reviews. Details of ongoing studies were sought through review of ClinicalTrials.Gov. There was no restriction on study locations. We restricted our inclusion criteria to studies only in the English language.

### Study selection and data collection

Two independent reviewers performed title, abstracts, full text screening, and data extraction. Data collection was performed using a standardized data collection form. We collected information regarding study design and enrollment criteria, baseline patient characteristics, and details of the intervention/comparator groups used in each study. Baseline patient characteristics included details on age, sex, background medical history, hemorrhage volume, start time of intervention, and clinical severity. Detailed outcome data on DVT and pulmonary embolism (PE) occurrence (asymptomatic/symptomatic, proximal/distal) was collected wherever possible. The primary outcome of interest was VTE, defined as the development of a proximal DVT (symptomatic or asymptomatic) or PE, diagnosed within the first 30 days of hemorrhage onset. The secondary outcome was the occurrence of any DVT (proximal or distal) or PE within the first thirty days. Safety data and treatment dropouts were reported where available.

### Risk of bias in individual studies

Two reviewers independently assessed the risk of bias of randomized controlled trials and non-randomized observational studies using the Cochrane Collaboration tool and the Robins-I, respectively [16,17]. Results were summarized in tabular format and to reduce the risk of bias in our final effect estimates, studies that scored “high risk” on a number of categories within the Cochrane risk of bias tool or “Critical/Serious” on the overall score of the Robins-I tool were excluded from our primary analysis.

### Data synthesis (Summary measures, synthesis of results)

We assessed baseline patient characteristics and event rates of the comparator arms to ensure that exchangeability assumptions were met and that there would be enough similarity between the included studies to allow for reliable data pooling [18]. We utilized an aggregate level Bayesian network meta-analyses to derive estimates of comparative effectiveness of each intervention relative to others in the treatment network. We calculated direct effect estimates between each intervention against a control. An indirect effect estimate was then calculated comparing the two interventions to each other using the control arm as a common comparator. Both outcomes of interest were reported in terms of dichotomous data, and findings from network-meta analyses were reported in terms of odds ratios (OR) with corresponding 95% credible intervals (CrIs). We performed all analyses using three chains of initial values, with totals of 50,000 or more burn-in and sampling iterations. Adequacy of model fit was assessed by comparing the posterior total residual deviance to the number of unconstrained data points in each analysis, and comparisons between models (for example, fixed versus random effects models) was based upon the deviance information criterion (DIC) (with differences of 5 points or more indicating an important difference). As neither of the networks studied included a closed loop, no checks for inconsistency of direct and indirect evidence were performed. We used the I^2^ statistic generated from traditional pairwise meta-analyses for each piece of the treatment network to assess statistical heterogeneity. We performed all analysis using RevMan 5.3 (Cochrane, London, UK), WinBUGS version 1.4.4 (MRC Biostatistics Unit, Cambridge, UK) and NetMetaXL packages [19].

### Risk of bias across studies and additional analyses

We compared all randomized controlled trials to protocols that had been previously published, if available. Pre-determined outcomes in the methodology were compared to the outcomes presented in the results section of each study report to monitor for changes in outcome prioritization and selective reporting. Funnel plots were planned to be assessed visually for indications of publication bias when 10 or more studies were available. We judged the quality of evidence for all outcomes using a framework developed by the GRADE working group specifically designed for randomized and non-randomized studies in the context of a network meta-analysis [20].

## Results

### Study selection

**Fig 1:**
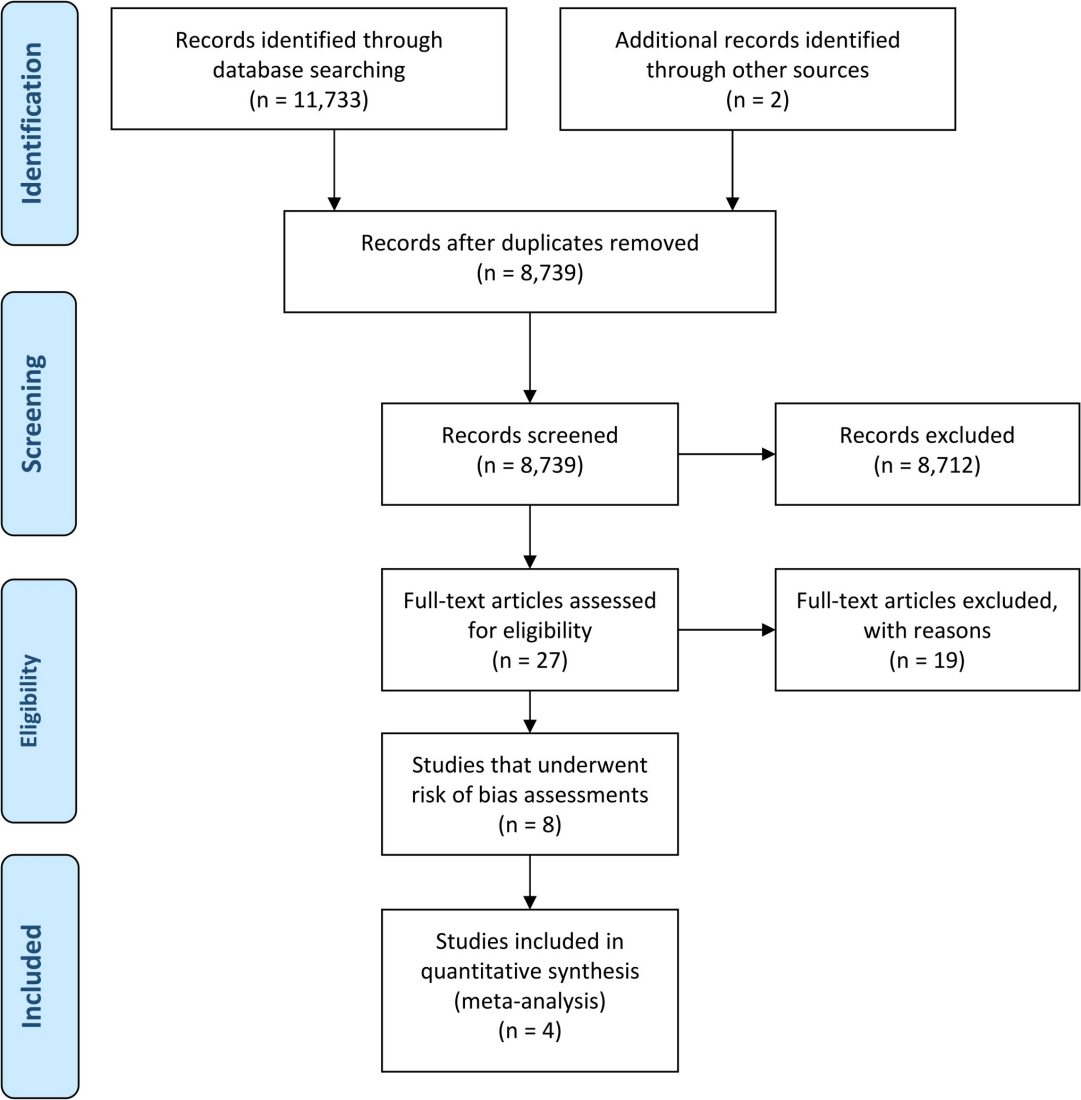
Literature search and study selection flow diagram.

Among 11,733 records retrieved by the literature search, there were 8,739 left for review after removal of duplicates across databases. Of these, 8,712 were excluded after screening of titles and abstracts, leaving 27 for full-text screening. Full-text screening resulted in exclusion of 19 studies (S2 Table). Eight studies, consisting of four randomized controlled trials and four non-randomized studies, were retained for inclusion in the review (Fig 1). The four non-randomized studies all scored severe/critical for overall risk of bias and were therefore excluded from primary analysis.

### Risk of bias within studies

Risk of bias assessments are summarized in S1 Fig and S3 Table. Risk of bias assessment deemed all RCTs appropriate for inclusion in the network meta-analysis. All four non-randomized studies scored highly for concerns of confounding and were deemed to possess a high risk of bias.

### Study characteristics

Of the four randomized controlled trials included in our analysis, one study focused on pharmacological treatment [21] and three focused on PCD [22–24]. Details of each study are included in Tables 1 and 2.

**Table 1:** Details of included studies (Pharmacological prophylaxis).

Study	Prevention of Deep Venous Thrombosis and Pulmonary Embolism in Patients With Acute Intracerebral Hemorrhage (Orken et al.[21])	
Year of Publication	2009	
Country(ies) of Study	Turkey	
Study Type	Randomized Controlled Trial	
Total Number of Patients	75	
Patient Characteristics	Intervention	Control
# of Patients	39	36
Age (mean, SD)	68.1 (11.98)	66.08 (9.6)
Male (n, %)	17 (43.6%)	28 (77.8%)
Hypertension	32 (82.1%)	29 (80.6%)
Prior Stroke /TIA	11 (28.2%)	6 (16.7%)
Alcohol Abuse	1 (2.6%)	3 (8.3%)
National Institute of Health Stroke Scale (mean, SD)	9.74 (6.06)	8.61 (6.87)
Location (n, %)		
Lobar (Subcortex)	11 (28.21%)	8 (22.22%)
Non-Lobar	28 (71.79%)	28 (77.78%)
Agent Used (Generic Name)	Enoxaparin Sodium	Long CS
Dose	40 mg	NA
Start Time from Hemorrhage Onset	48 Hours	48 Hours
Frequency of Dosing	Once Daily	NA
Route of Administration	Subcutaneous	NA
Duration of Treatment	21 days	21 days
VTE Events	4 (4 DVT, 0 PE)	4 (3 DVT, 1 PE)

Abbreviations: DVT = Deep vein thrombosis; ES = elastic stockings; CS = compression stockings; PCD = pneumatic compression device; PE = pulmonary embolism; NA = not applicable; NR = not recorded; SD = standard deviation

**Table 2:** Details of included studies (Pneumatic compression devices).

Study:	Prevention of Venous Thrombosis in Patients with Acute Intracerebral Hemorrhage (VICTORIAh)[24]		Intermittent pneumatic compression to prevent venous thromboembolism in patients with high risk of bleeding hospitalized in intensive care units: the CIREA1 randomized trial[23]		Effectiveness of intermittent pneumatic compression in reduction of risk of deep vein thrombosis in patients who have had a stroke (CLOTS 3): a multicentre randomized controlled trial[22]	
Year of Publication	2005		2013		2013	
Country(ies) of Study	France		France		United Kingdom	
Study Type	Randomized Controlled Trial		Randomized Controlled Trial		Randomized Controlled Trial	
Total Number of Patients	118		100		314	
Patient Characteristics	Intervention	Control	Intervention	Control	Intervention	Control
Number of Patients	53	65	56	44	156	158
Age (mean, SD)	63.2 (12.9)	67 (11.2)	57.6 (14.5)	56.4 (15.1)	72.4 (12.8)	72.8 (11.8)
Male (n, %)	32 (60.4)	35 (53.8)	30 (53.6)	27 (61.4)	84 (53.8%)	67 (57.6%)
Hypertension (n, %)	21 (39.6%)	34 (52.3%)	NR	NR	NR	NR
Cancer (n, %)	7 (13.2%)	1 (1.5%)	3 (5.4%)	2 (4.5%)	NR	NR
Antiplatelet Use (n, %)	9 (17%)	16 (24.6%)	7 (12.5%)	7 (15.9%)	7 (4.5%)	10 (6.3%)
Alcohol Abuse (n, %)	9 (24.5%)	13 (20.0%)	NR	NR	NR	NR
Glasgow coma scale (mean, SD)	9.5 (4.6)	9.4 (4.5)	NR	NR	NR	NR
Immobile (n, %)*	NR	NR	NR	NR	156 (100%)	158 (100%)
Neurosurgical Intervention (n, %)	14 (26.4%)	15 (23.0%)	16 (28.6%)	12 (27.3%)	NR	NR
Device Used	PCD + ES	ES	PCD + GCS	GCS	PCD	ES
Start Time from Hemorrhage Onset	<48 Hours	<48 Hours	<36 hours^	<36 hours*	Day 0–3	Day 0–3
Location: Knee/Thigh	Thigh-length	NA	Thigh-length	NA	Thigh-length	NA
Target Treatment Duration	10 Days	10 Days	6 Days	6 Days	30 Days	30 Days
Duration of Treatment (mean, SD)	8.5 (0.8)	8.5 (1.1)	5.9 (1.7)	6.1 (1.4)	15.0 (10.7)	NA
Treatment Dropouts (n,%)	9 (17%)	1 (1.5%)	2 (3.6%)	0 (0%)	2 (0.01%)	1 (0.003%)
VTE Events	4 (3 Distal DVT, 0 Proximal DVT, 1 PE)	11 (8 Distal DVT, 3 Proximal DVT, 0 PE)	4 (2 Distal DVT, 1 Proximal DVT, 1 PE)	6 (3 Distal DVT, 3 Proximal DVT, 0 PE)	34 (20 Distal DVT, 10 Proximal DVT, 4 PE)	46 (17 Distal DVT, 26 Proximal DVT, 3 PE)

*defined specifically by the CLOTS3 trialists as an inability to mobilize without help to the toilet

^within 36 hours of admission to ICU

Abbreviations: DVT = Deep vein thrombosis; ES = elastic stockings; GCS = graduated compression stockings; PCD = pneumatic compression device; PE = pulmonary embolism; NA = not applicable; NR = not recorded; SD = standard deviation

Orken et al. (2009) [21] was a single center randomized controlled trial where 75 patients with ICH were randomized to enoxaparin 40 mg versus compression stockings (control), 48 hours after stroke onset. Total duration of treatment was 21 days. Screening ultrasound and CT pulmonary angiography (CTPA) were performed on day 7. Repeat imaging was performed if patients were symptomatic from days 8–21. Lower extremity veins included the iliac, femoral, long saphenous, popliteal, and paired calf veins. For CTPA, any filling defects in the pulmonary arteries were interpreted as embolism. All radiological endpoints were evaluated in a blinded fashion. There were no treatment dropouts or complications reported by the investigators. Follow-up neuroimaging was performed at days 3, 7, and 21. Hematoma expansion was not observed in either treatment group.

The Venous Intermittent Compression and Thrombosis Occurrence Related to Intra-cerebral Acute hemorrhage study (VICTORIAh; 2005) [24] randomized patients with traumatic or spontaneous ICH to PCD or elastic stockings (control) at a single center. We were able to contact study authors to acquire summary outcome data on spontaneous ICH patients (n = 118). Patients were randomized within the first 48 hours of hospital admission and screening compression ultrasound was performed on day 10 by investigators blinded to treatment allocation. Day 10 screening ultrasound was used to determine the presence of asymptomatic lower limb DVT. Patients with symptoms of DVT at any time up to day 30 were evaluated by ultrasound. PE was diagnosed by high probability ventilation perfusion lung scan or CTPA, based on clinical suspicion from the treating physician. Information on hematoma expansion was not reported. Nine patients (17%) discontinued PCD therapy within the first 10 days due to lack of tolerance. Potential complications with PCDs was not reported.

The Compression pneumatique Intermittente en reanimation (CIREA1; 2013) [23] study was a multicenter, prospective, open label, blinded endpoint, randomized controlled trial comparing PCDs to graduation compression stockings (control) in intensive care unit patients within 48 hours of admission to the intensive care unit. Study authors were contacted to acquire summary outcome data on spontaneous ICH patients only (n = 100). Day 6 screening ultrasound was used to determine the presence of asymptomatic lower limb DVT. Patients with symptoms of DVT at any time up to day 30 were evaluated by ultrasound. Anticoagulation was contraindicated during the first six days of the study. Symptomatic PE within the first 30 days was assessed by CTPA based on clinical suspicion. Information on hematoma expansion was not reported. Two patients (3.6%) discontinued therapy due to lack of tolerance. PCD associated complications was not reported.

The Clots in Legs Or sTockings after Stroke (CLOTS3; 2013) [22] study was a multicenter trial in which immobilized acute stroke (ischemic or hemorrhagic) patients were randomized to PCDs plus standard care (which may include IV hydration, graduated compression stockings, antiplatelets, and anticoagulants) versus standard care alone. Patients were randomized within day 0–3 of stroke onset and screening compression ultrasound was performed on days 7–10 and days 25–30. The primary outcome was symptomatic or asymptomatic DVT in the popliteal or femoral (proximal) veins detected on a screening ultrasound or any symptomatic DVT in the popliteal or femoral veins, confirmed by imaging, within 30 days of randomization. Diagnosis of PE in the first 30 days was confirmed by CTPA or ventilation–perfusion (VQ) lung scan. Information on hematoma expansion was not reported. Data on hemorrhage characteristics was not collected. ICH patients (n = 376) were included in this study and individual patient data was acquired from study authors. The following patients were excluded: patients who were using anticoagulation prior to randomization (n = 4) or were started on anticoagulation following randomization for atrial fibrillation or an artificial heart valve (n = 4), and patients with missing outcome data or who died within the first 30 days (n = 54). PE was not identified as a cause of death within the first thirty days (S4 Table). Three-hundred and fourteen patients were included in our primary analysis. No skin breaks were observed in either treatment group.

Patient information from each study was compared and discussed. We judged that the exchangeability assumption for network meta-analysis was met and proceeded with our network meta-analyses.

### Synthesis of results

**Fig 2:**
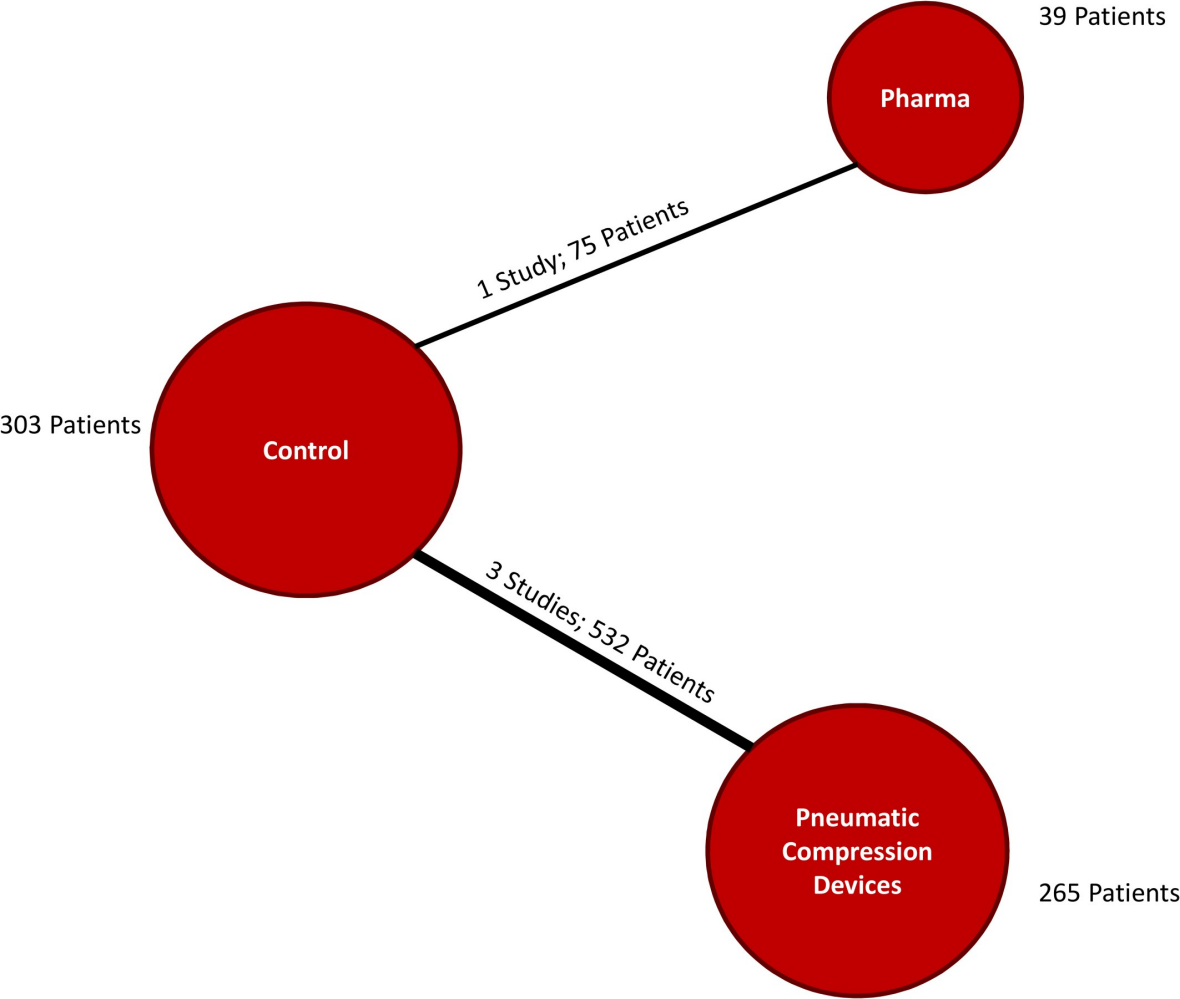
Network plots for pharmacotherapy (Pharma), pneumatic compression devices and venous thromboembolism. Each line connecting two nodes indicates a direct comparison and the thickness of each line is proportional to the number of trials directly comparing the two modalities.

**Fig 3:**
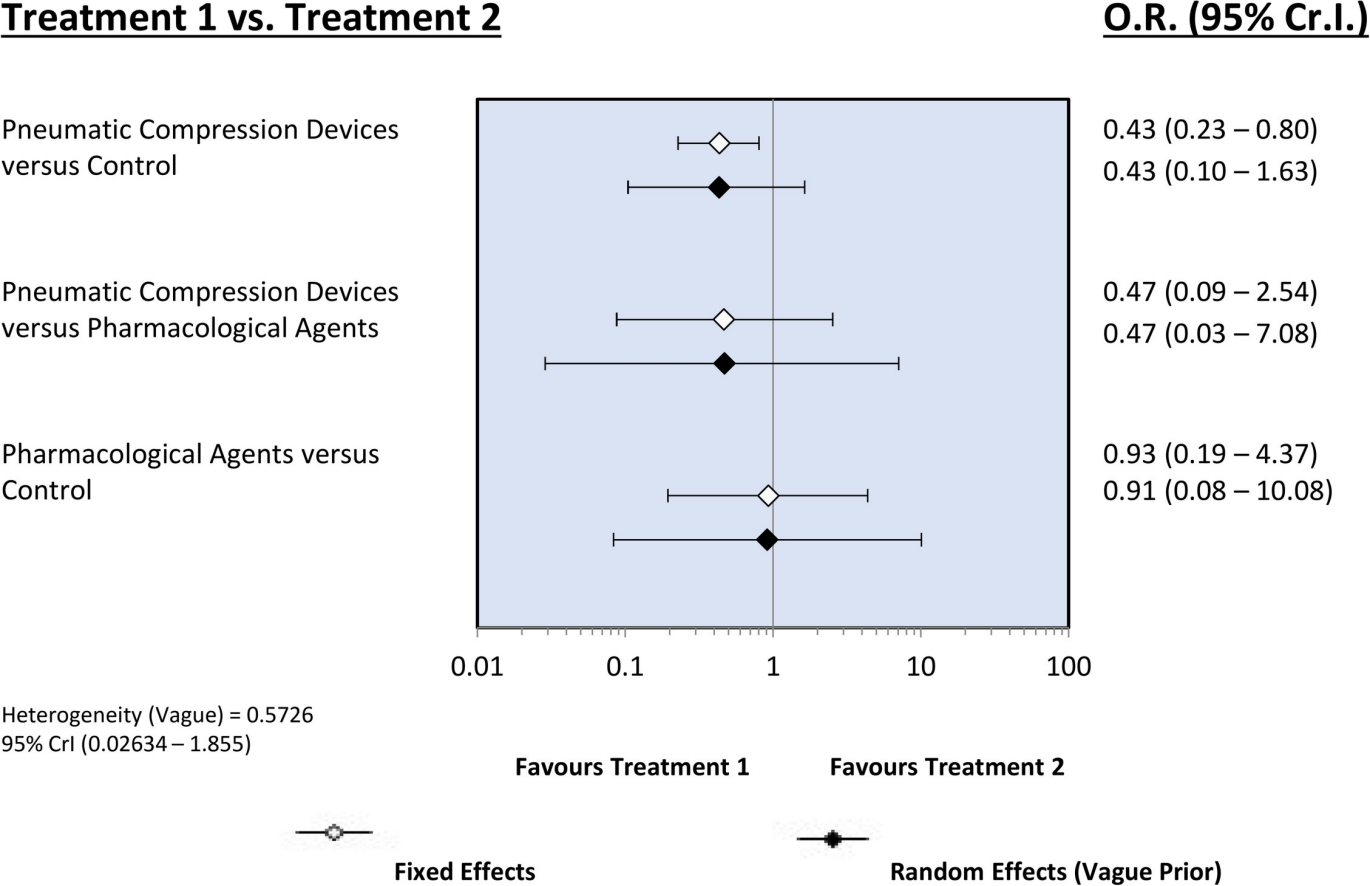
Network effect estimates forest plots for venous thromboembolism (Primary outcome). One direct comparison between pharmacotherapy and control. Three direct comparisons between pneumatic compression devices and control. 607 patients included in the analysis with 60 recorded events.

Head-to-head comparisons between pharmacotherapy and PCD from the included studies are depicted in the network plot seen in Fig 2. The overall incidence of VTE (proximal asymptomatic or symptomatic DVT, PE) was 9.9% (Tables 1 and 2). Assessment of measures of model fit (S5 Table) suggested reasonable fit of both the fixed effects (FE) and random effects (RE) models and DIC estimates were similar. Direct and indirect comparisons are summarized in Fig 3. Findings from the FE analysis suggested that PCDs are associated with a significantly decreased odds of venous thromboembolism compared to control interventions (OR: 0.43, 95% CrI: 0.23–0.80). No clear difference between pharmacological thromboprophylaxis and control was observed (OR: 0.93, 95% CrI: 0.19–4.37). On indirect assessment, no significant difference between PCD and pharmacological therapy was found (OR: 0.47, 95% CrI: 0.09–2.54). A similar pattern of findings was noted when data on *any DVT/PE* was assessed (S2 Fig).

CLOTS3 was the sole study to collect data on delays to treatment initiation. When stratified by initiation time, patients started on PDCs within days 0–1 benefited to a statistically significant degree (PCD: 7/74, 9.5% versus Control: 15/68, 22.1%; p<0.05), compared to patients who started treatment on days 2–3 (PCD: 7/82, 8.5% versus Control: 14/90, 15.5%; p = 0.160). A meta-analysis of hematoma expansion and mortality was dropped as data on these secondary outcomes were largely not reported.

### Risk of bias across studies and strength of evidence

Two of the four randomized controlled trials were registered (CIREA1, CLOTS3). The outcomes and study methodology outlined in the protocol match with the final manuscript. For the remaining randomized controlled trials, the pre-determined outcome measures matched with the final results reported.

Randomized trials formed the evidence base for both pharmacotherapy and PCD assessments (S6 Table). Imprecision with inclusion of the null value resulted in the overall quality of network meta-analysis to be considered “moderate” overall.

## Discussion

While guidelines support the use of VTE prophylaxis in acute ICH, data around the relative efficacy of management options are lacking [4,5,11,25,26]. In this systematic review, we used a network meta-analysis to compare PCDs to pharmacological thromboprophylaxis.

The total event rate in our network was 9.9%, which is in keeping with previous studies of symptomatic DVT rates in ICH varying from 3 to 7% [2,27]. In direct comparison, PCD were superior to control interventions. This comparison is supported by three high quality randomized controlled trials [22–24]. Each study assessed a mixed population of patients and as such, we felt it prudent to contact study authors and seek out data on ICH patients, alone. The generated effect estimates are in keeping with a recently performed review [28], however, we strengthened our assessment by acquiring ICH patient data from all three trials, thereby significantly reducing cohort and outcome heterogeneity.

Compression devices studies aimed to randomize patients within the 48–72 hours of symptom onset, whereas pharmacotherapy was started only after the 48-hour mark. In CLOTS3, 45% of patients were randomized on day 0–1, while 55% were randomized on day 2–3. Our post-hoc assessment of these sub-groups suggests that a delay in treatment reduces the overall effectiveness of compression devices. It stands to reason that the potential risk of VTE may be front-loaded. Analysis of the CLOTS 1 and 2 datasets reported the majority of DVT events occurring in the first seven days from stroke onset [29]. In addition, an observational study of pharmacotherapy by Boeer et al. [30] reported that the majority of VTE events were observed at a day two ultrasound. This may partially explain the neutral results reported by Orken et al. [21], where pharmacological agents were only introduced 48 hours after symptom onset. However, our comparative analysis is limited by the inclusion of only one study evaluating pharmacotherapy and the neutral findings observed may be secondary to a limited study sample.

Although we assessed a second randomized study performed by Dickmann et al. [31] for potential inclusion in our primary analysis, a treatment start-time of four days post hemorrhage occurrence did not meet entry criteria based on our a priori protocol; this delay to treatment start was not reflective of present practice in most institutions, or in line with practice guidelines [4,5]. We also considered including observational studies, as was done by previous reviews on pharmacotherapy [32], but issues with significant confounding and overall methodology precluded us from doing so. Ultimately, meaningful conclusions regarding the effectiveness, timing, or duration of anticoagulation cannot be made from the results of a single study and randomized controlled trials are required to evaluate this further.

Additional limitations include a relative lack of reporting of safety data, mortality, and disability, with CLOTS3 being the sole study to provide a robust assessment of these measures. The absence of this data reflects areas of must needed improvement and highlights the need for further investigations in pharmacological prophylaxis and the greater need for increased safety data with either intervention.

## Conclusions

Early initiation of PCD is associated with a lower odds of VTE in patients presenting acutely with ICH when compared to control interventions. A relative lack of data around pharmacotherapy limits the conclusions we can make from our network meta-analysis and further questions should be explored via large pragmatic trials.

## Supporting information

S1 Checklist PRISMA NMA checklist of items to include when reporting a systematic review involving a network meta-analysis. (DOCX)

S1 Table Sample search strategy. (PDF)

S2 Table Summary of excluded studies after full-text screening. (PDF)

S3 Table Risk of bias assessment (Non-randomized studies). (PDF)

S4 Table Baseline characteristics of excluded patients in the CLOTS3 cohort. (PDF)

S5 Table Summary model Fits of NMA. (PDF)

S6 Table Quality of evidence assessment (GRADE). (PDF)

S1 Fig Risk of bias assessments (Randomized controlled trials). (PDF)

S2 Fig Network meta-analysis:–Any DVT/PE. (PDF)
