# Supplementary material for: Venous thromboembolism prevention in intracerebral hemorrhage: A systematic review and network meta-analysis
Source: PLoS One. 2020 Jun 24;15(6):e0234957. doi: 10.1371/journal.pone.0234957 (PMC7314010; doi:10.1371/journal.pone.0234957)
Supplement: S1 Table — (PDF) [file pone.0234957.s002.pdf]

Supplement Table 1: Sample Search Strategy

**Search Terms for Pneumatic Compression Devices**		**Search Terms for Pharmacologic Prophylaxis**	
1.	MeSH Terms: cerebral hemorrhage/ or basal ganglia hemorrhage/ or putaminal hemorrhage/ or cerebral intraventricular hemorrhage/	27.	MeSH Terms: dalteparin/ or enoxaparin/
2.	MeSH Terms: intracranial hemorrhages/ or intracranial hemorrhage, hypertensive/	28.	MeSH Terms: heparin/ or heparin, low-molecular-weight/
3.	(intracerebral adj2 h?emorrhage*).tw.	29.	lovenox.tw.
4.	(h?emorrhagic adj2 stroke*).tw.	30.	heparin*.tw.
5.	(cerebral adj3 h?emorrhage*).tw.	31.	dalteparin.tw.
6.	(putam* adj2 h?emorrhage).tw.	32.	tedelparin*.tw.
7.	(intracerebral adj2 h?ematoma).tw.	33.	fragmin*.tw.
8.	(basal gangl* adj2 h?emorrhage).tw.	34.	(FR860 or FR 860).tw.
9.	(intraventricular adj2 h?emorrhage).tw.	35.	(kabi 2165 or kabi2165).tw.
10.	(intraventricular adj2 h?ematoma).tw.	36.	EMT*.tw.
11.	(basal gangl* adj2 h?ematoma).tw.	37.	enoxaparin*.tw.
12.	(ICH*).tw.	38.	clexane.tw.
13.	(intracerebral adj3 bleed*).tw.	39.	PK10*.tw.
14.	1 or 2 or 3 or 4 or 5 or 6 or 7 or 8 or 9 or 10 or 11 or 12 or 13	40.	liquaemin.tw.
15.	MeSH Terms: Intermittent Pneumatic Compression Devices/	41.	(pharmacological adj3 prophylaxis).tw.
16.	(pneumatic adj3 device*).tw.	42.	(systemic adj3 prophylaxis).tw.
17.	(pneumatic adj3 compression*).tw.	43.	(antithromb* or anti-thromb*).tw.
18.	(pneumatic adj3 stocking*).tw.	44.	fondaparinux.tw.
19.	(pneumatic adj3 hose*).tw.	45.	apixaban.tw.
20.	(mechanical adj2 compression).tw.	46.	rivaroxaban.tw.
21.	Sequential Compression Device.tw.	47.	dabigatran.tw.
22.	(mechanical adj3 prophylaxis).tw.	48.	(short-term adj3 anticoagulation).tw.
23.	(calf adj3 compression).tw.	49.	27 or 28 or 29 or 30 or 31 or 32 or 33 or 34 or 35 or 36 or 37 or 38 or 39 or 40 or 41 or 42 or 43 or 44 or 45 or 46 or 47 or 48
24.	calf pump.tw.	50.	14 and 49
25.	15 or 16 or 17 or 18 or 19 or 20 or 21 or 22 or 23 or 24		
26.	14 and 25		
