# Supplementary material for: Venous thromboembolism prevention in intracerebral hemorrhage: A systematic review and network meta-analysis
Source: PLoS One. 2020 Jun 24;15(6):e0234957. doi: 10.1371/journal.pone.0234957 (PMC7314010; doi:10.1371/journal.pone.0234957)
Supplement: S2 Table — (PDF) [file pone.0234957.s003.pdf]

**Supplement Table 2: Summary of Excluded Studies After Full-Text Screening:**

| Authors | Title | Journal | Year | Reason for Exclusion |
|---|---|---|---|---|
| Abhay et al.[1] | Incidence of Deep Venous Thrombosis (DVT) related to type of DVT prophylaxis in Patients with Intracerebral Haemorhrage | Journal of Clinical Neuroscience | 2009 | Abstract Only. Report contains insufficient information to assess methodological quality |
| Cherian et al.[2] | Current Practice Trends for Use of Early Venous Thromboembolism Prophylaxis After Intracerebral Hemorrhage | Neurosurgery | 2018 | Limited information on primary outcome (VTE incidence) |
| Chibbaro et al.[3] | Safety of Deep Venous Thrombosis Prophylaxis with Low-Molecular Weight Heparin in Brain Surgery. Prospective Study of 746 Patients | Surgical Neurology | 2008 | Single cohort study with no comparative group |
| Chibbaro et al.[4] | Evolution of Prophylaxis Protocols for Venous Thromboembolism in Neurosurgery: Results from a Prospective Comparative Study on Low-Molecular-Weight Heparin, Elastic Stockings, and Intermittent Pneumatic Compression Devices. | World Neurosurgery | 2018 | Heterogenous Population (tumors, hemorrhage). Intracerebral hemorrhage populations could not be isolated out. |
| Dickmann et al.[5] | Heparin therapy, deep-vein thrombosis and pulmonary embolism after intracerebral hemorrhage | Klinische Wochenschrift | 1988 | Precursor study of Boeer et al. which included an additional cohort with a prophylaxis start time of < 48 hours. |
| Frontera et al.[6] | Comparison of pharmacologic versus mechanical venous thromboemoblism prophylaxis in patients with intracranial hemorrhage | Stroke | 2014 | Abstract Only. Report contains insufficient information to assess methodological quality |
| Frontera et al.[7] | Safety of venous thromboembolism prophylaxis in intracranial hemorrhage patients with external ventricular drains. | Stroke | 2014 | Abstract Only. Report contains insufficient information to assess methodological quality |
| Li et al.[8] | DVT prophylaxis after ICH in China: Results from china national stroke registry (CNSR). | Stroke | 2011 | Abstract Only. Report contains insufficient information to assess methodological quality |
| Khripun et al.[9] | Safety and efficacy of various regimens of heparin-mediated prevention of venous thromboses in patients with intracranial haemorrhage | Angiology and vascular surgery | 2012 | Manuscript in the Russian Language |
| Masotti et al.[10] | Pharmacological prophylaxis of venous thromboembolism in patients with spontaneous intracerebral hemorrhage. | Italian Medical Journal | 2013 | Abstract Only. Report contains insufficient information to assess |
| Masotti et al.[11] | Venous thromboembolism prevention in spontaneous intracerebral hemorrhage: Preliminary results of a protocol based on sequential strategy of intermittent pneumatic compression followed by low molecular weight heparin. | Italian Medical Journal | 2016 | 1) Abstract Only. Report contains insufficient information to assess methodological quality.<br>2) Single cohort with no comparative group |

| | | | | |
|---|---|---|---|---|
| Munoz-Venturelli et al.[12] | Prophylactic Heparin in Acute Intracerebral Hemorrhage: A Propensity Score-Matched Analysis of the INTERACT2 Study | International Journal of Stroke | 2016 | Did not include information on the primary outcome (DVT/PE or Hematoma Expansion) |
| Nurmohamed et al.[13] | Low molecular weight heparin and compression stockings in the prevention of venous thromboembolism in neurosurgery | Thrombosis and Haemostasis | 1996 | 1) Data could not be acquired<br>2) Unclear patient population |
| Orken et al.[14] | Heparin treatment for the proflaxis of deep venous thrombosis in the subacute stage of intracerebral hemorrhages | Turk Beyin Damar Hastaliklar Dergisi. | 2007 | 1) Manuscript in the Turkish Language<br>2) Abstract (English Translation) indicates that this was a single cohort population with no comparative arm. |
| Phelan et al.[15] | A randomized, double blinded, placebo-controlled pilot trial of anticoagulation in low-risk traumatic brain injury: The Delayed Versus Early Enoxaparin Prophylaxis I (DEEP I) Study | Journal of Trauma and Acute Care Surgery | 2012 | Patient population exclusive to traumatic brain injury. No inclusion of spontaneous intracerebral hemorrhage. |
| Shah et al.[16] | Early venous thromboembolism (VTE) prophylaxis in spontaneous intracerebral hemorrhage (SICH) | Neurocritical Care | 2017 | 1) Abstract Only. Report contains insufficient information to assess methodological quality |
| Wen & Hall[17] | Complications of subcutaneous low-dose heparin therapy in neurosurgical patients. | Surgical Neurology | 1998 | Ineligible population |
| Zurasky et al.[18] | DVT prophylaxis in intracerebral hemorrhage patients in a large health system: A 5 year analysis. | Stroke | 2016 | Abstract Only. Report contains insufficient information to assess methodological quality |
| Sprugel et al.[19] | Heparin for prophylaxis of venous thromboembolism in intracerebral haemorrhage. | Journal of Neurology, Neurosurgery, and Psychiatry | 2019 | Lack of comparator arm |

**References**:

1. Venkhat A, Hughes A, Lueck C. Incidence of Deep Venous Thrombosis (DVT) related to type of DVT Prophylaxis in Patients with Intracerebral Haemorrhage. *J. Clin. Neurosci.* [Internet]. 2009;16:1542. Available from: http://linkinghub.elsevier.com/retrieve/pii/S0967586809004950

2. Cherian LJ, Smith EE, Schwamm LH, Fonarow GC, Schulte PJ, Xian Y, et al. Current Practice Trends for Use of Early Venous Thromboembolism Prophylaxis After Intracerebral Hemorrhage. *Neurosurgery* [Internet]. 2018;82:85–92. Available from: http://www.ncbi.nlm.nih.gov/pubmed/28379461

3. Chibbaro S, Tacconi L. Safety of deep venous thrombosis prophylaxis with low-molecular-weight heparin in brain surgery. Prospective study on 746 patients. *Surg. Neurol.* [Internet]. 2008;70:117–21; discussion 121. Available from: http://www.ncbi.nlm.nih.gov/pubmed/18262633

4. Chibbaro S, Cebula H, Todeschi J, Fricia M, Vigouroux D, Abid H, et al. Evolution of Prophylaxis Protocols for Venous Thromboembolism in Neurosurgery: Results from a Prospective Comparative Study on Low-Molecular-Weight Heparin, Elastic Stockings, and Intermittent Pneumatic Compression Devices. *World Neurosurg.* [Internet]. 2018;109:e510–e516. Available from: http://www.ncbi.nlm.nih.gov/pubmed/29033376

5. Dickmann U, Voth E, Schicha H, Henze T, Prange H, Emrich D. Heparin therapy, deep-vein thrombosis and pulmonary embolism after intracerebral hemorrhage. *Klin. Wochenschr.* [Internet]. 1988;66:1182–3. Available from: http://www.ncbi.nlm.nih.gov/pubmed/3062268

6. Frontera JA, Jovine M, Hunter S, Catalano A, Gordon E. Abstract T MP83: Comparison of Pharmacologic versus Mechanical Venous Thromboemoblism Prophylaxis in Patients with Intracranial Hemorrhage. *Stroke* [Internet]. 2014;45:ATMP83 LP-ATMP83. Available from: http://stroke.ahajournals.org/content/45/Suppl_1/ATMP83.abstract

7. Frontera JA, Jovine M, Zach V, Gordon E. Abstract T P236: Safety of Venous Thromboembolism Prophylaxis in Intracranial Hemorrhage Patients with External Ventricular Drains. *Stroke* [Internet]. 2014;45:ATP236 LP-ATP236. Available from: http://stroke.ahajournals.org/content/45/Suppl_1/ATP236.abstract

8. Li H.; Li Z.; Mai N.-H.; Zhao X.; Wang C.; Zhou Y.; Liu L.; Wang Y. DVT prophylaxis after ICH in China: Results from china national stroke registry (CNSR). *Stroke* [Internet]. 2011;42:e111 LP-e350. Available from: http://stroke.ahajournals.org/content/42/3/e111.abstract

9. Khripun AN, Shurygin SN, Priamikov AD, Mironkov AB, Asratian SA, Petrenko N V, et al. [Safety and efficacy of various regimens of heparin-mediated prevention of venous thromboses in patients with intracranial haemorrhage]. *Angiol. Sosud. Khir.* [Internet]. 2012;18:20–5. Available from: http://www.ncbi.nlm.nih.gov/pubmed/22836324

10. Masotti L.; Gori S.; Bellizzi A.; Ubaldi E.; Fenu P.; Cannistraro D.; Mannucci A.; Scotto

F.P.; Gianchecchi D.; Bini C.; Corchia A.; Pampana A. Pharmacological prophylaxis of venous thromboembolism in patients with spontaneous intracerebral hemorrhage. *Ital. J. Med.* 2013;7:78.

11. Masotti L.; Seravalle C.; Vannucchi V.; Moroni F.; Pallini F.; Pesci A.; Chiarelli R.; Landini G. Venous thromboembolism prevention in spontaneous intracerebral hemorrhage: Preliminary results of a protocol based on sequential strategy of intermittent pneumatic compression followed by low molecular weight heparin. *Ital. J. Med.* 2016;10:74.

12. Muñoz-Venturelli P, Wang X, Lavados PM, Stapf C, Robinson T, Lindley R, et al. Prophylactic heparin in acute intracerebral hemorrhage: a propensity score-matched analysis of the INTERACT2 study. *Int. J. Stroke* [Internet]. 2016;11:549–56. Available from: http://www.ncbi.nlm.nih.gov/pubmed/27009893

13. Nurmohamed MT, van Riel AM, Henkens CM, Koopman MM, Que GT, D'Azemar P, et al. Low molecular weight heparin and compression stockings in the prevention of venous thromboembolism in neurosurgery. *Thromb. Haemost.* [Internet]. 1996;75:233–8. Available from: http://www.ncbi.nlm.nih.gov/pubmed/8815566

14. Necioglu Orken D.; Kenangil G.; Guner C.; Celik M.; Forta H. Primer intraserebral kanamali hastalarda derin ven trombozu proflaksisinde heparin kullanimi. *Turk Beyin Damar Hast. Dergisi.* 2007;13:37–39.

15. Phelan HA, Wolf SE, Norwood SH, Aldy K, Brakenridge SC, Eastman AL, et al. A randomized, double-blinded, placebo-controlled pilot trial of anticoagulation in low-risk traumatic brain injury: The Delayed Versus Early Enoxaparin Prophylaxis I (DEEP I) study. *J. Trauma Acute Care Surg.* [Internet]. 2012;73:1434–41. Available from: http://www.ncbi.nlm.nih.gov/pubmed/22914079

16. Shah, Sheikh, Au, Sullivan, Rincon V. Early venous thromboembolism (VTE) prophylaxis in spontaneous intracerebral hemorrhage (SICH). *Neurocrit. Care*. 2017;27:S134.

17. Wen DY, Hall WA. Complications of subcutaneous low-dose heparin therapy in neurosurgical patients. *Surg. Neurol.* [Internet]. 1998;50:521–5. Available from: http://www.ncbi.nlm.nih.gov/pubmed/9870811

18. Zurasky JF, Stuchiner T, Baraban E. Abstract WP363: DVT Prophylaxis in Intracerebral Hemorrhage Patients in a Large Health System: a 5 Year Analysis. *Stroke* [Internet]. 2016;47:AWP363 LP-AWP363. Available from: http://stroke.ahajournals.org/content/47/Suppl_1/AWP363.abstract

19. Sprügel MI, Sembill JA, Kuramatsu JB, Gerner ST, Hagen M, Roeder SS, et al. Heparin for prophylaxis of venous thromboembolism in intracerebral haemorrhage. *J. Neurol. Neurosurg. Psychiatry* [Internet]. 2019;90:783–791. Available from: http://jnnp.bmj.com/lookup/doi/10.1136/jnnp-2018-319786

20. Boeer A, Voth E, Henze T, Prange HW. Early heparin therapy in patients with spontaneous intracerebral haemorrhage. *J. Neurol. Neurosurg. Psychiatry* [Internet]. 1991;54:466–7. Available from: http://www.ncbi.nlm.nih.gov/pubmed/1865215

21. Wasay M, Khan S, Zaki KS, Khealani BA, Kamal A, Azam I, et al. A non-randomized study of safety and efficacy of heparin for DVT prophylaxis in intracerebral haemorrhage. *J. Pak. Med. Assoc.* [Internet]. 2008;58:362–4. Available from: http://www.ncbi.nlm.nih.gov/pubmed/18988406

22. Tetri S, Hakala J, Juvela S, Saloheimo P, Pyhtinen J, Rusanen H, et al. Safety of low-dose subcutaneous enoxaparin for the prevention of venous thromboembolism after primary intracerebral haemorrhage. *Thromb. Res.* [Internet]. 2008;123:206–12. Available from: http://www.ncbi.nlm.nih.gov/pubmed/18420258

23. Levy AS, Salottolo K, Coplin WM, Smith R, Santos P, Bar-Or D. Abstract 208: Pharmocologic Thromboprophylaxis Reduces the Odds of Venous Thromboembolism in Patients With Hemorrhagic Stroke. *Stroke* [Internet]. 2014;45:A208 LP-A208. Available from: http://stroke.ahajournals.org/content/45/Suppl_1/A208.abstract
