# Supplementary material for: Venous thromboembolism prevention in intracerebral hemorrhage: A systematic review and network meta-analysis
Source: PLoS One. 2020 Jun 24;15(6):e0234957. doi: 10.1371/journal.pone.0234957 (PMC7314010; doi:10.1371/journal.pone.0234957)
Supplement: S3 Table — (PDF) [file pone.0234957.s004.pdf]

Supplement Table 3: Risk of Bias Assessments of Non-Randomized Studies

Study Author	Confounding	Selection	Intervention Classification	Intervention Deviation	Missing Data	Measurement of Outcomes	Reported Result	Overall
Tetri et al. 2008	Serious	Serious	Moderate	Low	Low	Serious	NI	**Serious**
Wasay et al. 2008	Serious	Moderate	Moderate	Low	Low	Serious	NI	**Serious**
Boeer et al. 1991	Serious	Serious	Low	Low	Low	Low	NI	**Serious**
Levy et al. 2014	Serious	Low	Moderate	Low	Low	Serious	NI	**Serious**

Abbreviations: NI=information not available

Boeer et al.[1] was a single center non-randomized controlled study and is an extension of a prior randomized controlled trial, first conducted in 1988.[2] Forty-five acute intracerebral hemorrhage patients received either heparin (5000 units, started within 48 hours of hemorrhage onset) or no treatment. Phleboscintigraphy and pulmonary perfusion scans were performed on day 10. A description of how the exposed cohort was selected was not provided. Baseline patient characteristics were also not extensively reported.

Wasay et al. (2008)[3] was a retrospective cohort study of 458 patients. Patients received either heparin (started between days 1 to 7 post hemorrhage event) or compression stockings at the discretion of the treating physician. Daily clinical assessments were performed to assess for deep vein thrombosis and leg ultrasounds were used for confirmation. Patients were reportedly matched for age, sex, diabetes, and smoking status, but the underlying techniques were not described and significant differences in premorbid functioning were observed between the two groups. Outcome assessments were not blinded.

Tetri et al. (2008)[4] was a single center retrospective cohort study of 407 patients. Patients were given enoxaparin if they suffered limb paralysis or immobility. Only patients with some degree of immobility were allocated to receiving pharmacotherapy. Control populations had more mobility at baseline and were not provided any treatment. No confounding adjustment was described. Intervention start times varied widely between study subjects. Clinical suspicion of a thrombotic complication led to imaging (ultrasound) to confirm. Outcome assessments were not blinded.

Levy et al (2014)[5] was a single center retrospective cohort study of 243 patients. Patients received either pharmacologic thromboprophylaxis, mechanical compression, or a combination of the two. The exact type of mechanical compression was not defined and the exact pharmacological agent varied. All DVT events were symptomatic and outcome assessments were not blinded.

References:

1. Boeer A, Voth E, Henze T, Prange HW. Early heparin therapy in patients with spontaneous intracerebral haemorrhage. *J. Neurol. Neurosurg. Psychiatry* [Internet]. 1991;54:466–7. Available from: http://www.ncbi.nlm.nih.gov/pubmed/1865215

2. Dickmann U, Voth E, Schicha H, Henze T, Prange H, Emrich D. Heparin therapy, deep-vein thrombosis and pulmonary embolism after intracerebral hemorrhage. *Klin. Wochenschr.* [Internet]. 1988;66:1182–3. Available from: http://www.ncbi.nlm.nih.gov/pubmed/3062268

3. Wasay M, Khan S, Zaki KS, Khealani BA, Kamal A, Azam I, et al. A non-randomized study of safety and efficacy of heparin for DVT prophylaxis in intracerebral haemorrhage. *J. Pak. Med. Assoc.* [Internet]. 2008;58:362–4. Available from: http://www.ncbi.nlm.nih.gov/pubmed/18988406

4. Tetri S, Hakala J, Juvela S, Saloheimo P, Pyhtinen J, Rusanen H, et al. Safety of low-dose subcutaneous enoxaparin for the prevention of venous thromboembolism after primary intracerebral haemorrhage. *Thromb. Res.* [Internet]. 2008;123:206–12. Available from: http://www.ncbi.nlm.nih.gov/pubmed/18420258

5. Levy AS, Salottolo K, Coplin WM, Smith R, Santos P, Bar-Or D. Abstract 208: Pharmocologic Thromboprophylaxis Reduces the Odds of Venous Thromboembolism in Patients With Hemorrhagic Stroke. *Stroke* [Internet]. 2014;45:A208 LP-A208. Available from: http://stroke.ahajournals.org/content/45/Suppl_1/A208.abstract
