# Supplementary material for: Venous thromboembolism prevention in intracerebral hemorrhage: A systematic review and network meta-analysis
Source: PLoS One. 2020 Jun 24;15(6):e0234957. doi: 10.1371/journal.pone.0234957 (PMC7314010; doi:10.1371/journal.pone.0234957)
Supplement: S4 Table — (PDF) [file pone.0234957.s005.pdf]

Supplement Table 4: Baseline Characteristics of Excluded Patients in the CLOTS3 Cohort

**Patient Characteristics**	**N=62**
Age (mean, SD)	76.6 (11.7)
Male (n, %)	32 (51.6)
Antiplatelet use (n, %)	6 (9.7)
Any DVT at 30 days (n, %)	3 (4.8)
Cause of Death (n, %)	
Neurological Damage From Initial Hemorrhage	21 (33.9)
Pneumonia	14 (22.6)
Pulmonary Embolism	0 (0.0)
Recurrent Stroke	5 (8.1%)
Coronary Heart Disease	1 (1.6%)
