# Supplementary material for: Venous thromboembolism prevention in intracerebral hemorrhage: A systematic review and network meta-analysis
Source: PLoS One. 2020 Jun 24;15(6):e0234957. doi: 10.1371/journal.pone.0234957 (PMC7314010; doi:10.1371/journal.pone.0234957)
Supplement: S5 Table — (PDF) [file pone.0234957.s006.pdf]

Supplement Table 5: Model fits from NMA, Proximal DVT/PE

Model	# unconstrained data points	Posterior Total residual deviance	SD	DIC
FE consistency	8	6.21	NA	38.29
RE consistency	8	6.95	0.57	39.70

Adequate model fit was considered to be present if the posterior total residual deviance was comparable to the # of unconstrained data points. Fit between different models was considered similar when DIC were within 5 points of each other for the models under consideration.
