# Supplementary material for: Venous thromboembolism prevention in intracerebral hemorrhage: A systematic review and network meta-analysis
Source: PLoS One. 2020 Jun 24;15(6):e0234957. doi: 10.1371/journal.pone.0234957 (PMC7314010; doi:10.1371/journal.pone.0234957)
Supplement: S6 Table — (PDF) [file pone.0234957.s007.pdf]

Supplement Table 6: Quality of Evidence Assessment Using GRADE (Fixed Effects)

**Comparison**	**Direct Evidence**		**Indirect Evidence**		**Network Meta-Analysis**	
	Odds Ratio (95% CrI)	Quality of Evidence	Odds Ratio (95% CrI)	Quality of Evidence	Odds Ratio (95% CrI)	Quality of Evidence
Pharmacotherapy v. Control	0.93 (0.19-4.37)	Moderate*	-	-	0.93 (0.19-4.37)	Moderate*
PCD v. Control	0.43 (0.23-0.80)	High^	-	-	0.43 (0.23-0.80)	High^
PCD v. Pharmacotherapy	-	-	0.47 (0.09-2.54)	Moderate$	0.47 (0.09-2.54)	Moderate$

*Randomized trials form the evidence base and the quality rating starts with high. Concerns of imprecision due to 95% CrI involving the null value

^ Randomized trials form the evidence base and the quality rating starts with high.

$Randomized trials form the evidence base and the quality rating starts with high. Concerns of imprecision due to 95% CrI involving the null value
