# Supplementary material for: Venous thromboembolism prevention in intracerebral hemorrhage: A systematic review and network meta-analysis
Source: PLoS One. 2020 Jun 24;15(6):e0234957. doi: 10.1371/journal.pone.0234957 (PMC7314010; doi:10.1371/journal.pone.0234957)
Supplement: S1 Fig — (PDF) [file pone.0234957.s008.pdf]

Supplement Figure 1: Risk of Bias Assessments (Randomized Controlled Trials)

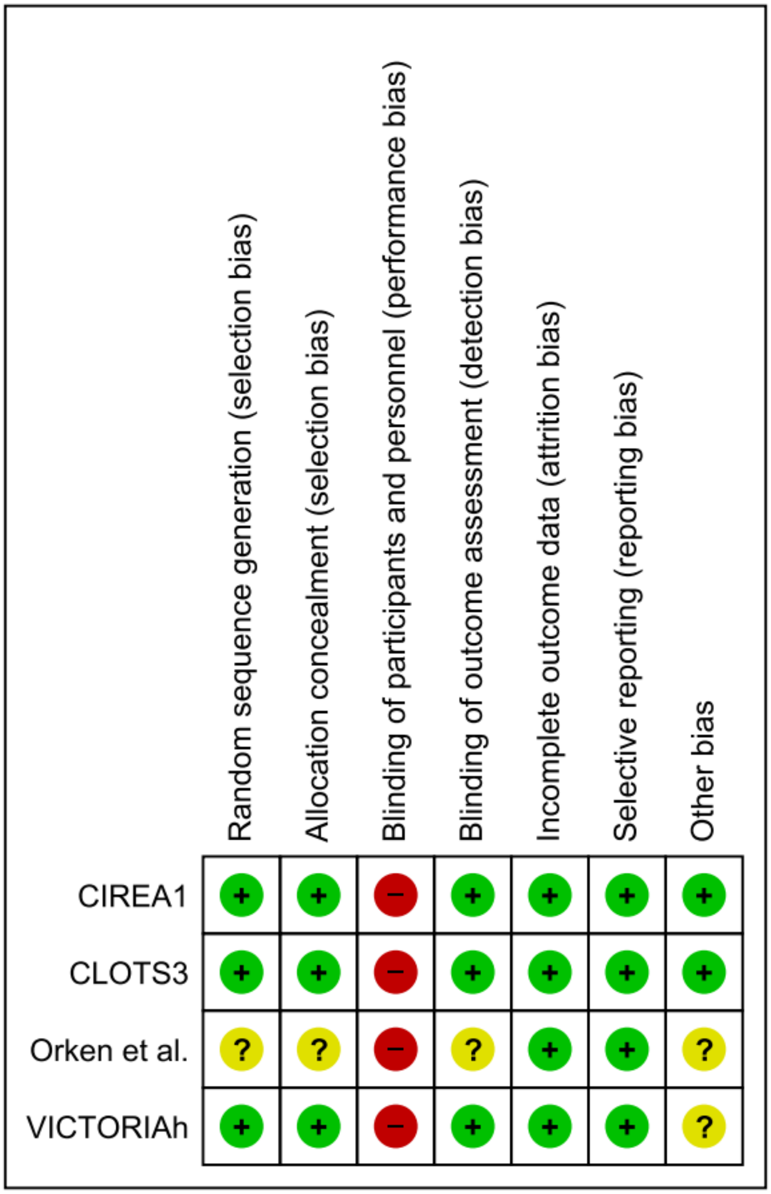

Green = Low Risk of Bias

Yellow = Risk of Bias Unclear

Red = High Risk of Bias
