# Supplementary material for: Venous thromboembolism prevention in intracerebral hemorrhage: A systematic review and network meta-analysis
Source: PLoS One. 2020 Jun 24;15(6):e0234957. doi: 10.1371/journal.pone.0234957 (PMC7314010; doi:10.1371/journal.pone.0234957)

Supplement Figure 2: Network Effect Estimates Forest Plots for Venous Thromboembolism (Any DVT/PE). One direct comparison between pharmacotherapy and control. Three direct comparison between pneumatic compression devices and control. 607 patients included in the analysis with 112 recorded events.

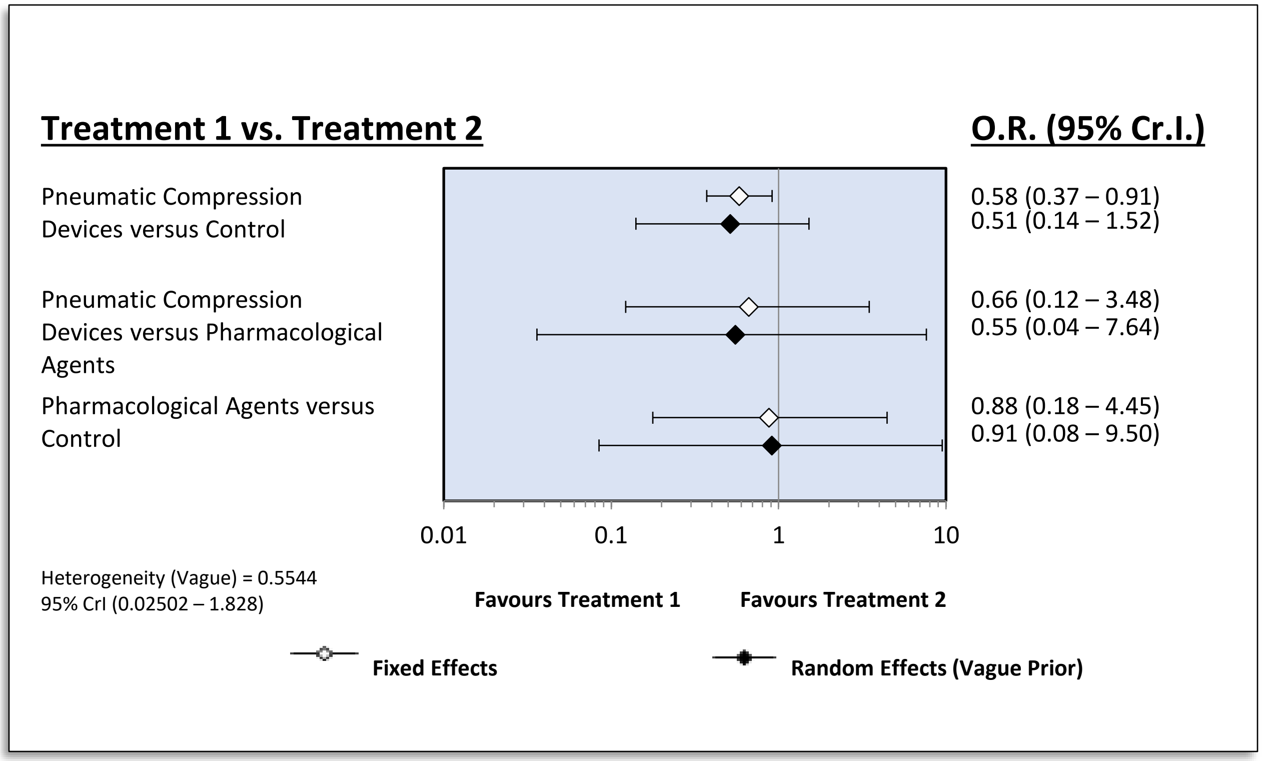

Supplement: S2 Fig — (PDF) [file pone.0234957.s009.pdf]
